# Winter Grazing in Vineyards Suppresses Pathogens and Promotes Grapevine Health

**DOI:** 10.3390/plants15060864

**Published:** 2026-03-11

**Authors:** Shaowei Cui, Lianzhu Zhou, Dong Li, Yanni Song, Hui Wu, Xiaoqing Huang, Decai Jin, Haijun Xiao, Yongqiang Liu

**Affiliations:** 1 School of Grassland Science, Beijing Forestry University, Beijing 100083, China; shaowei_cui@126.com (S.C.); lidong318@bjfu.edu.cn (D.L.); songyanni@bjfu.edu.cn (Y.S.); 2 State Key Laboratory for Biology of Plant Diseases and Insect Pests, Institute of Plant Protection, Chinese Academy of Agricultural Sciences, Beijing 100193, China; pillar1017@126.com (L.Z.); wuhuii0919@126.com (H.W.); huangxiaoqing0718@126.com (X.H.); 3 Research Center for Eco-Environmental Sciences, Chinese Academy of Sciences, Beijing 100085, China; dcjin@rcees.ac.cn

**Keywords:** integrated system, sheep grazing, winter sanitation, grape health, microbial communities

## Abstract

Crop residues can harbor pathogens, making winter sanitation essential for sustainable viticulture. The grass–sheep–grape system could improve vineyard health through microbial optimization. To evaluate this, we assessed the effects of sheep feeding on fallen leaves on the occurrence of grape diseases through greenhouse experiments and used high-throughput-sequencing to compare microbial communities in grape fallen leaves and sheep feces, aiming to determine whether winter grazing reduces residue-borne pathogens. The results revealed that sheep grazing in vineyards significantly reduces the occurrence of grape leaf and cluster diseases, as well as a fundamental difference in microbial structures between leaves and feces, with no fungal taxa detected in the feces. The number of shared bacterial OTUs was minimal, while feces contained significantly more unique bacterial OTUs than fallen leaves. Additionally, bacterial diversity was significantly higher in feces than in fallen leaves. Sheep feces harbored a substantial number of highly efficient cellulose-degrading anaerobic bacteria, which may enhance organic matter conversion efficiency, and promote nutrient cycling in vineyards. Moreover, the grazing process directly reduced several pathogenic fungi associated with grape leaf, fruit, and root diseases. Functional analysis further indicated that fecal bacterial communities were primarily enriched in core metabolic and genetic processing functions, while leaf microbes were more involved in microbial interactions and secondary metabolism. More importantly, no function guilds of plant pathogenic fungi were present in feces. Overall, winter sheep grazing in vineyards can remove fallen leaves, not only reducing the risk of pathogen transmission but also potentially introducing beneficial bacterial communities. This study provides a feasible strategy for organic vineyard management in winter, and offers important insights for promoting sustainable vineyard production.

## 1. Introduction

Crop residues, including fallen leaves, straw, and root stubble, are generally considered waste. Retaining crop residues in fields through agricultural practices such as no-till farming play a crucial role in reducing greenhouse gas emissions, mitigating soil degradation, conserving moisture, sequestering carbon, remediating pollutants, fostering beneficial microorganisms, and improving soil structure and overall health, thereby enhancing plant productivity [1]. This practice has been widely adopted globally [2]. However, crop residues can serve as reservoirs for diverse microbial communities, including certain plant pathogens, thereby increasing the risk of disease transmission and epidemics [2,3]. Furthermore, some pathogenic fungi can enter a dormant state under unfavorable conditions and germinate when conditions become suitable in the following year, posing a threat to the health of subsequent crops [4]. This is of particular importance for perennial crops.

Therefore, in agricultural management, sanitation refers to cultural control practices that involve removing and destroying pathogen-infested plant materials, crop residues, and other pathogen inoculum sources from fields and surrounding areas [5]. This practice can significantly reduce the initial pathogen inoculum, and prevent their spread within and between cropping seasons, thereby lowering the incidence or delaying the onset of diseases [5,6]. Removing crop residues is a fundamental and highly effective component of integrated pest management. From both economic and ecological perspectives, effective sanitation practices are low-cost, proactive measures that can reduce dependence on pesticides, minimize the risk of pesticide resistance in pest populations, lessen environmental pollution, and promote the overall health of farmland ecosystems. However, due to growing concerns over environmental protection and costs, conventional methods such as manual removal and burning are no longer optimal choices.

Grapevine (*Vitis vinifera* L.), is one of the most widely cultivated and economically important fruit crops globally, with substantial production and consumption worldwide [7]. China ranks among the leading countries in both vineyard area and grape production, underscoring the crop’s major agricultural and economic significance nationally [8]. Grape leaves host diverse microbial communities, including beneficial microorganisms that support vine growth, health, productivity, and wine quality characteristics [9,10,11]. However, pathogenic microbes including powdery mildew, *Botrytis cinerea*, and *Plasmopara viticola* and so on, colonize leaf surfaces and threaten vine health and production sustainability [12,13,14]. Fallen leaves that remain in the vineyard can serve as refugia for pathogens, increasing the risk of disease infection in the following growing season. As a perennial crop, this may directly threaten the sustainable production of vineyards.

Grazing livestock in croplands is a common practice for resource utilization and field management [15,16]. The integrated grass–sheep–grape system represents an innovative ecological agriculture practice that incorporates livestock into the agroecosystem. Building on the use of cover crops in vineyard inter-rows, this system introduces grazing sheep as biological regulators of forage biomass. Concurrently, sheep can consume pruned shoots and leaves and help remove fallen leaves during winter in the vineyard, contributing to enhanced agricultural sustainability [17]. This integrated approach further enhances resource-use efficiency, reduces reliance on chemical inputs, and can generate supplementary income [16]. It also promotes soil nutrient cycling, and creates favorable conditions for biodiversity conservation, and the maintenance of ecosystem functions [18,19]. Moreover, such integrated agroforestry systems can strengthen ecosystem services, enhance interactions within the agricultural ecosystem, improve economic sustainability, and increase resilience to climatic disturbances [20,21].

Integrated crop-livestock systems can provide greater ecological and economic benefits compared to traditional viticultural practices [22]. Recent studies suggest that the grass–livestock–fruit system can support grapevine growth, health, and productivity by modulating microbial communities, and notably grazing has been shown to enrich beneficial microbes while significantly reducing the abundance of pathogenic microorganisms in the vineyard [17]. Nevertheless, the mechanisms driving these microbial shifts, particularly the reduction in pathogens, remain unclear. Therefore, this study investigated the impact of sheep feeding on fallen leaves on grape disease occurrence through a greenhouse experiment. High-throughput sequencing was also used to compare the microbial communities in fallen leaves and sheep feces. The goal was to determine whether winter grazing reduces crop-residue-borne pathogens, offering insights for sustainable vineyard management.

## 2. Materials and Methods

### 2.1. Experimental Design

The experiment was conducted in a grape greenhouse in Binzhou, Shandong Province, China (118°9′ E, 37°1′ N). The region has a temperate continental monsoon climate, with an average annual temperature of 13.6 °C, and precipitation of 582.8 mm. The grape cultivar grown was ‘Shine Muscat’. In the experimental vineyard, ryegrass was planted in mid-October 2022, followed by sheep grazing treatment (GS) starting in March 2023, with 12 sheep grazing for 10 days each month for three consecutive years. Meanwhile, sheep are grazed in vineyards during winter to remove fallen leaves. A clean tillage vineyard served as the control (CK). The experimental vineyard followed conventional irrigation and fertilization, while disease and pest management relied on plant-derived pesticides and biocontrol agents to ensure sheep safety.

In late September of 2024 and 2025, leaf and cluster diseases were investigated in both the treatment and control groups. Disease severity was graded according to previously established criteria [17]. The grading scales were as follows, for leaves: Grade 0: Healthy leaf, no lesions; grade 1: Lesion area < 5%; grade 3: Lesion area 6–25%; grade 5: Lesion area 26–50%; grade 7: Lesion area 51–75%; grade 9: Lesion area > 76%. For clusters: Grade 0: Healthy cluster, no infected berries; grade 1: Diseased berries < 5%; grade 3: Diseased berries 6–15%; grade 5: Diseased berries 16–25%; grade 7: Diseased berries 26–50%; grade 9: Diseased berries > 51%. The rate of diseased leaves (RDL, %) and the rate of diseased clusters (RDC, %) were calculated as follows: RDL (%) = (number of diseased leaves/total leaves) × 100; RDC (%) = (number of diseased clusters/total clusters) × 100. The disease index (DI) was calculated using the following formula: DI = [Σ (disease grade × number of leaves or clusters in that grade)/(total number of leaves or clusters × 9)] × 100.

**Figure 1:**
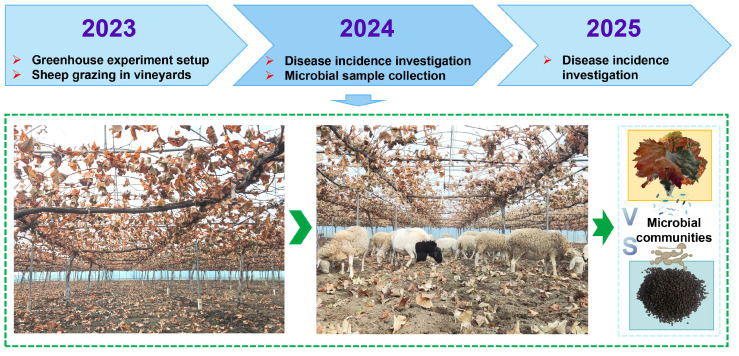
Experimental design of sheep-assisted vineyard sanitation during winter. Grass was planted in mid-October 2022, followed by sheep grazing from March 2023 for three consecutive years. Leaf and fruit disease incidence was assessed in late September of 2024 and 2025, and leaf and fecal samples were collected in December 2024 for microbial analysis.

In November 2024, the vineyard soil was plowed, in early December wilted grape leaves were manually knocked down. Seven sampling sites were established in an “S” shape throughout the vineyard, and five fallen leaves were randomly collected from each site. Twelve adult sheep were then introduced into the greenhouse and allowed to freely feed on the fallen leaves for 24 h. Then, seven sheep were randomly selected thereafter, and fresh fecal samples were collected. The experimental design is illustrated in Figure 1. Fallen leaf and fecal samples were placed in insulated boxes, kept chilled, transported to the laboratory within 12 h, and stored at −80 °C pending subsequent analyses.

### 2.2. DNA Extraction and Sequencing of Fallen Leaf and Fecal Samples

First, the collected fallen leaves and sheep fecal samples were freeze-dried using a freeze-dryer (Model: FD-1C-50; Beijing Boyikang Experimental Instrument Co., Ltd, Beijing, China). The samples were then placed in a mortar, and liquid nitrogen was added for thorough grinding. Subsequently, 0.5 g of the ground leaf and fecal samples were weighed, and total DNA was extracted using the FastDNA^®^ Spin Kit for Soil (Cat. No.: 6560200; Manufacturer: MP Biomedicals, Irvine, CA, USA). The16S rRNA gene was amplified using primers 799F (5′-AACMGGATTAGATACCCKG-3′), and 1115R (5′-AGGGTTGCGCTCGTTG-3′), and the ITS region was amplified with primers gITS7F (5′-GTGARTCATCGARTCTTTG-3′) and ITS4 (5′-TCCTCCGCTTATTGATATGC-3′). The PCR reaction systems and conditions, the purification of PCR products, and high-throughput sequencing were performed following the methods described by previous research [8].

### 2.3. Microbial Characterization and Data Analysis

Raw sequencing data was pre-processed on the Galaxy platform (https://dmap.denglab.org.cn) [23]. Paired-end reads were merged using FLASH, and low-quality sequences were filtered out. Chimeric sequences were removed, and operational taxonomic units (OTUs) were clustered using UPARSE. Taxonomic assignment was performed using the RDA training set No. 19 (RDA Classifier 2.14 database) for bacteria and the UNITE 8.3 database for fungi.

To assess microbial community structure, principal coordinate analysis (PCoA) based on Bray–Curtis distance was conducted, and permutational multivariate analysis of variance (PERMANOVA) was used to test for significant differences among groups. Community composition was characterized at both the phylum and genus levels.

Functional profiles of bacterial and fungal communities were predicted using Tax4Fun2 and FUNGuild, respectively [24,25]. The overall workflow for microbial data analysis followed previous studies [8].

All statistical analyses were conducted using SPSS Statistics 27 software (IBM, Armonk, NY, USA). The rate of diseased leaves or clusters, disease index, microbial alpha diversity indices, relative abundances at the phylum and genus, and functional guild were tested for normality, and then analyzed using independent sample *t*-tests. Differences were considered statistically significant at *p* < 0.05.

## 3. Results

### 3.1. Incidence of Leaf and Cluster Diseases

**Figure 2:**
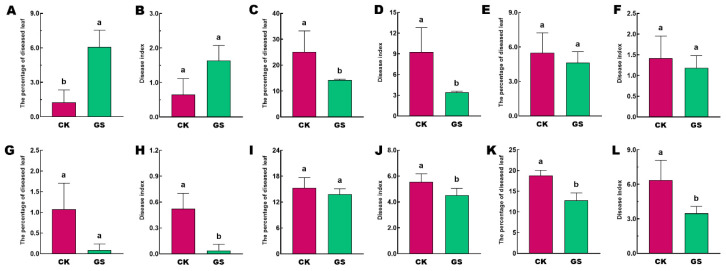
Incidence of leaf diseases in grapevines. (**A**–**F**): The survey shows results from the second year of treatment; (**G**–**L**): Results from the third year. (**A**,**B**,**G**,**H**) represent downy mildew; (**C**,**D**,**I**,**J**) represent leaf spot; (**E**,**F**,**K**,**L**) represent sooty blotch. Data are shown as mean ± SE, different letters denote significant differences at *p* < 0.05.

Sheep grazing in vineyards significantly reduced the incidence of grapevine leaf diseases. Compared to the clean tillage (CK), grazing treatment (GS) significantly decreased both the incidence and disease index of leaf spot (Figure 2C,D,I,J). For downy mildew, in the second year of treatment, the disease incidence in the GS treatment remained significantly higher than that in CK (Figure 2A). However, after three years of treatment, GS significantly reduced the occurrence of the disease, with both disease incidence and disease index being significantly lower in GS than in CK (Figure 2G,H). Regarding sooty blotch, no significant difference was observed between treatments in the second year (Figure 2E,F), but in the third year, GS resulted in a significantly lower incidence and disease index compared to CK (Figure 2K,L).

**Figure 3:**
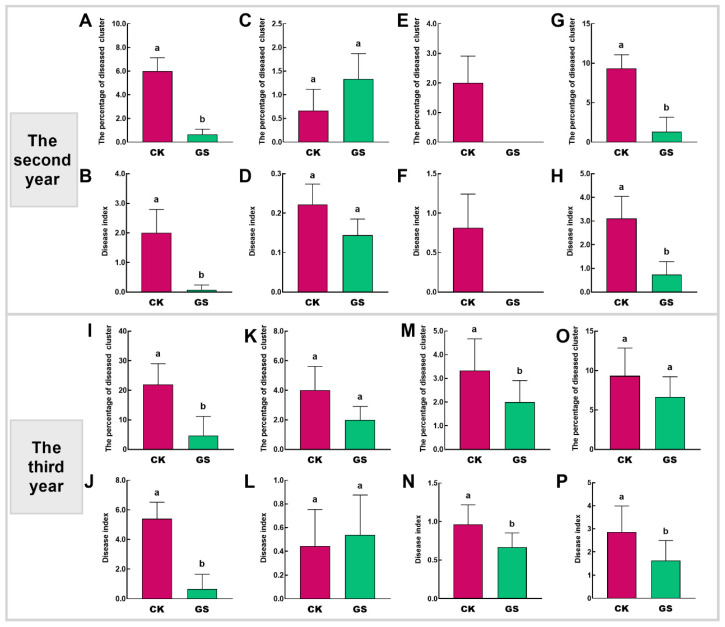
Incidence of grape cluster diseases. (**A**,**B**,**I**,**J**): Gray mold; (**C**,**D**,**K**,**L**): Ripe rot; (**E**,**F**,**M**,**N**): Sour rot; (**G**,**H**,**O**,**P**): *Aspergili*. Data are shown as mean ± SE, different letters denote significant differences at *p* < 0.05.

As shown in Figure 3, compared with the CK treatment, the GS treatment significantly reduced the incidence and disease index of grape cluster gray mold, sour rot, and *Aspergili* in both the second and third years. Overall, sheep grazing in vineyards significantly reduced the occurrence of grape leaf and cluster diseases, and this treatment exhibited a cumulative effect.

### 3.2. Analysis of Microbial Diversity in Grape Leaf and Sheep Feces

**Figure 4:**
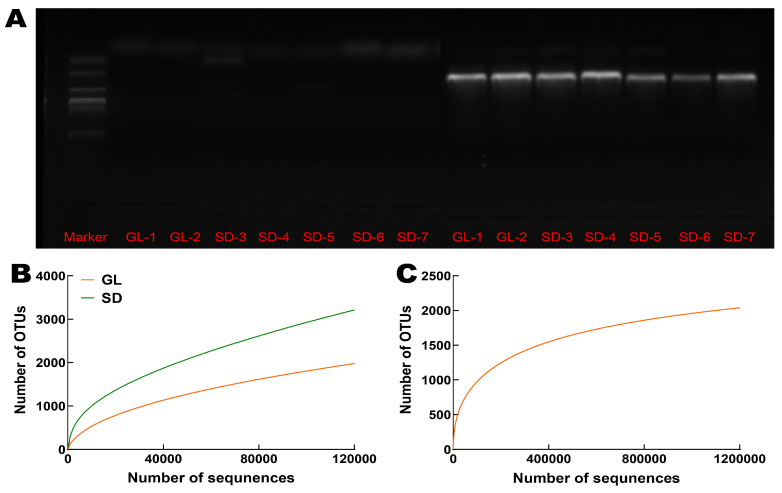
Electrophoresis results of fungal PCR products (**A**) and rarefaction curves for bacteria (**B**) and fungi (**C**).

A total of 1,342,342 high-quality bacterial sequences and 1,340,700 high-quality fungal sequences were obtained from leaf litter and fecal samples. After data normalization and resampling, 8831 bacterial and 2073 fungal reads were retained for subsequent analysis. Notably, no fungal ITS amplicons were obtained from the sheep fecal samples despite repeated PCR amplifications (Figure 4A; the original electrophoresis gel images are shown in Figures S1 and S2), suggesting that the viable fungal community was largely eliminated, or its DNA was reduced below the detection threshold. Rarefaction curves reached a plateau, indicating that the sequencing depth adequately captured the microbial features of both leaf and fecal samples (Figure 4B,C).

**Figure 5:**
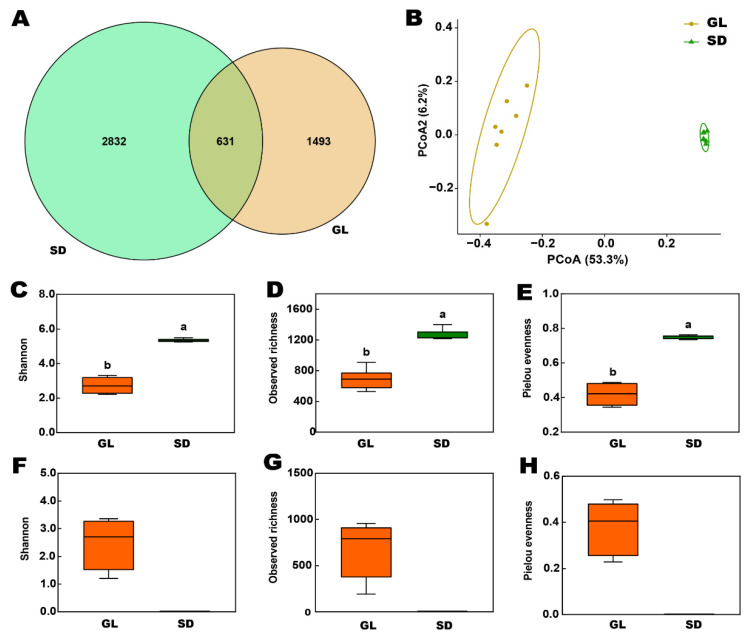
Diversity analysis of fallen leaf and fecal samples. (**A**): The OTU distribution of bacterial communities in fallen leaf and feces; (**B**): The beta diversity analysis of bacterial communities; (**C**–**E**): The alpha diversity of bacterial communities; (**F**–**H**): The alpha diversity of fungal communities; (**C**–**H**): Data are shown as mean ± SE, different letters denote significant differences at *p* < 0.05.

Categorical analysis revealed that fallen leaf (GL) and fecal (SD) samples shared 631 OTUs, with fecal samples harboring a significantly higher number of unique OTUs (2832) compared to leaf samples (Figure 5A). Principal coordinate analysis (PCoA) revealed that PCoA1 explained 53.53% of bacterial variation, while PCoA2 accounted for 6.2%, with distinct separation between fallen leaf and fecal bacterial communities (Figure 5B). PERMANOVA analysis revealed extremely significant differences between fallen leaf and fecal bacterial communities (F = 13.47, R^2^ = 0.53, *p* < 0.001). Alpha diversity analysis indicated significant differences in bacterial and fungal community alpha diversity between fallen leaf and feces. Fecal bacterial communities exhibited the highest Shannon, observed richness, and Pielou evenness, with all alpha diversity indices significantly exceeding those of leaf bacteria. For fungal communities, alpha diversity was present in fallen leaf fungal communities, whereas there were no fungal distribution in fecal samples.

### 3.3. Leaf and Feces Microbiome Composition

**Figure 6:**
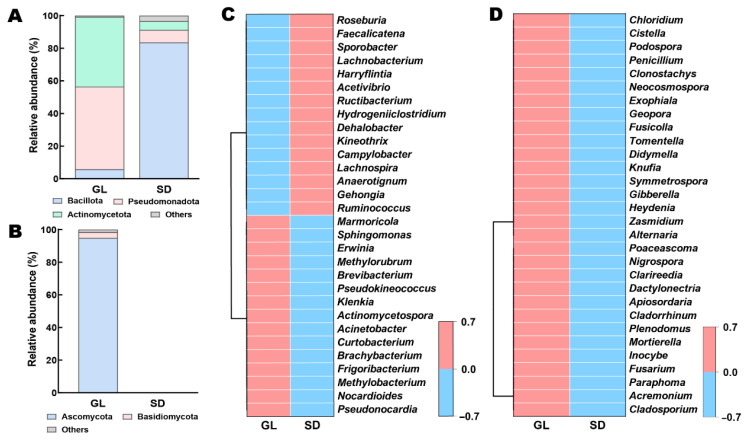
Composition of bacterial (**A**,**C**) and fungal (**B**,**D**) communities in fallen leaf and sheep feces samples at the phylum and genus levels.

The dominant bacterial phyla were Bacillota (5.87–83.61%), Pseudomonadota (7.68–50.61%), and Actinomycetota (5.36–42.76%) (Figure 6A). Among them, the relative abundance of Bacillota was significantly higher in feces than in fallen leaf (*p* < 0.001), whereas Pseudomonadota and Actinomycetota were significantly more abundant in fallen leaf than in feces (*p* < 0.001). For fungal communities, Ascomycota and Basidiomycota were the dominant phyla, both detected only in fallen leaf and absent in feces (Figure 6B).

The top 30 bacterial genera are shown in Figure 6C. In fallen leaf, *Pseudonocardia*, *Methylorubrum*, *Methylobacterium*, *Frigoribacterium*, *Erwinia*, *Nocardioides*, *Curtobacterium*, *Acinetobacter*, *Actinomycetospora*, *Klenkia*, *Sphingomonas*, *Pseudokineococcus*, and *Brevibacterium* were significantly more abundant. Among these, *Methylobacterium*, *Frigoribacterium*, *Brachybacterium*, *Klenkia*, and *Pseudokineococcus* were not detected in sheep feces. In contrast, sheep feces showed significantly higher relative abundances of *Ruminococcus*, *Hydrogeniiclostridium*, *Gehongia*, *Campylobacter*, *Kineothrix*, *Dehalobacter*, *Ructibacterium*, *Acetivibrio*, *Harryflintia*, *Lachnobacterium*, *Sporobacter*, *Faecalicatena*, *Anaerotignum*, *Lachnospira* and *Roseburia*.

Figure 6D presents the top 30 fungal genera, all of which were exclusively detected in leaf litter samples. Notably, several pathogenic genera closely associated with grapevine leaf, cluster, and root diseases, such as *Alternaria*, *Fusarium*, *Gibberella*, *Didymella*, *Cladosporium*, *Nigrospora*, *Zasmidium*, *Paraphoma*, *Neocosmospora*, *Dactylonectria* and *Clarireedia*. But these genera were absent in fecal samples.

Overall, sheep consumption of fallen leaf directly reduced pathogen groups closely associated with grapevine leaf, cluster, and root diseases. Meanwhile, sheep feces introduced a substantial amount of ruminant gut symbionts and fermentative bacteria into the vineyard, which may accelerate nutrient cycling and enhance soil fertility.

### 3.4. Functional Profiling of Microbial Communities

**Figure 7:**
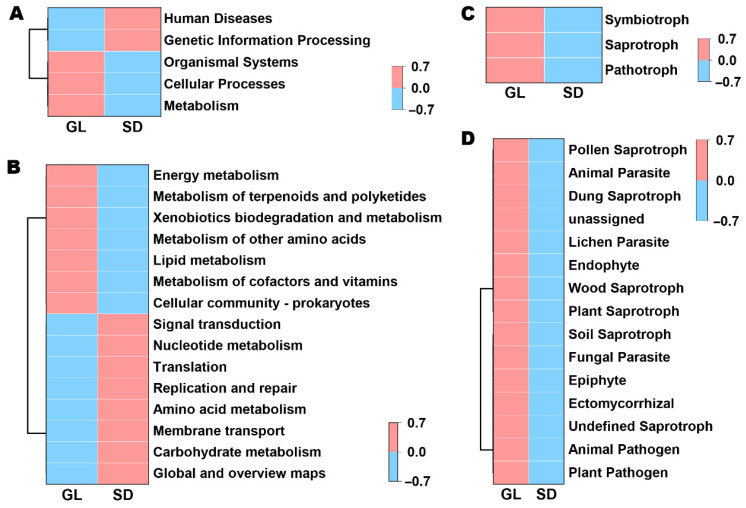
Functional analysis of bacterial (**A**,**B**) and fungal (**C**,**D**) communities in fallen leaves and sheep feces samples.

Functional prediction results for the bacterial community (Figure 7A,B) indicate that metabolism, genetic information processing, and cellular processes were the dominant functional categories. Among these, the relative abundances of metabolism and organismal systems were significantly higher in fallen leaf samples than in fecal samples (*p* < 0.001). In contrast, genetic information processing was significantly more abundant in feces (*p* < 0.05). Regarding specific bacterial functional pathways, categories such as global and overview maps, carbohydrate metabolism, amino acid metabolism, membrane transport, signal transduction, nucleotide metabolism, translation, and replication and repair were enriched in fecal samples. However, pathways including cellular community-prokaryotes, metabolism of cofactors and vitamins, lipid metabolism, xenobiotics biodegradation and metabolism, metabolism of terpenoids and polyketides, and metabolism of other amino acids showed higher relative abundances in fallen leaf.

For the fungal community, functional trophic modes included pathotroph, saprotroph, and symbiotroph, all of which were exclusively detected in fallen leaf (Figure 7C). Furthermore, the functional category of plant pathogen, which is closely associated with plant health, was present only in fallen leaf samples, and not detected in feces (Figure 7D). This further indicates that sheep grazing on fallen leaves can reduce pathogen-associated groups in the vineyard ecosystem.

## 4. Discussion

Plant residues, such as fallen leaves, can provide overwintering refuges for pathogens, thereby increasing the risk of disease transmission and epidemics. Introducing sheep into vineyards during winter can help remove grapevine fallen leaves. However, the impact of sheep grazing in vineyards on the occurrence of grape diseases, as well as whether grazing can effectively eliminate pathogens on fallen leaves, remains unclear. Therefore, this study clarified the impact of sheep grazing on grape disease occurrence in a greenhouse experiment and employed high-throughput sequencing to analyze the microbial structure and function of grapevine fallen leaf and the feces of sheep after consuming the grape fallen leaves.

Grazing can alter the structure and composition of soil microbial communities, thereby influencing ecosystem functions [26]. Sheep grazing in vineyards, as an increasingly adopted cover crop management practice, helps reduce fertilizer inputs, promotes carbon and nitrogen cycling, and enhances soil health [16]. Additionally, grazing contributes to biodiversity conservation and the maintenance of ecological functions [19]. Recent studies have shown that the integrated grass–sheep–grape system can enrich beneficial microorganisms and suppress pathogenic fungi by modulating microbial communities, thereby promoting grapevine growth and health [17]. This study revealed that sheep grazing in vineyards significantly reduces the incidence of leaf and cluster diseases in grapevines. This finding aligns with previous research showing that grazing can reduce plant leaf spot diseases by directly or indirectly regulating the composition and structure of microbial communities, which play a crucial role in maintaining plant health [27]. Similarly, in vineyard systems, sheep consumption of fallen leaves directly removes pathogen-carrying residues, disrupts overwintering sites for pathogens, and thereby reduces primary inoculum pressure in the following growing season. Notably, the disease suppression effect became more pronounced in the third year of treatment, suggesting a cumulative effect of grazing on disease regulation. This may be attributed to the sustained shaping of soil and phyllosphere microbial communities by long-term grazing, as well as the gradual establishment and stabilization of beneficial microbial populations [17].

Microbial analysis revealed that there is a fundamental difference in microbial composition between fallen leaves and sheep feces. Notably, no fungal taxa were detected in the feces, and very few OTUs were shared between the two sample types. Feces contained a large number of unique bacterial OTUs, indicating that the microbial community underwent strong selection] and restructuring after ingestion and passage through the digestive tract. The ruminant digestive system acts as a stringent environmental filter, unfavorable for the survival of epiphytic and saprotrophic leaf-associated microbes [28,29]. We also found that the alpha diversity of the bacterial community (including Shannon, richness, and evenness index) was significantly higher in sheep feces compared to grape fallen leaves. This can be primarily attributed to the diverse micro-niches and complex substrates provided by the ruminant digestive process, which selectively enriches unique bacterial taxa [30,31]. The ingested material serves as a substrate for the re-colonization and proliferation of resident gut microbiota, ultimately leading to higher microbial diversity in feces than in the original litter [28,29]. However, our findings show that fungal taxa were undetectable in fecal samples, implying that the viable fungal community was largely eliminated or its DNA was reduced below the detection limit after passage through the sheep’s digestive system. This is likely the result of combined effects during ingestion and digestion, including physical disruption (e.g., mastication), exposure to rumen microbiota and enzymes, prolonged anaerobic rumen fermentation, and passage through the acidic abomasum and proteolytic small intestine. Although fungal survival may vary by species and environment, many saprotrophic and pathogenic fungi are not adapted to long-term survival in the ruminant gut [32]. This aligns with previous findings that bacteria typically constitute the majority of microbial biomass in the rumen, though the intake of fresh feed can also increase the abundance and diversity of other microorganisms [33].

Microbial analysis indicated that grapevine leaf litter harbors several fungal pathogens, including species of *Alternaria*, *Fusarium*, *Gibberella*, *Didymella* and *Cladosporium*. Additionally, our survey results revealed that in the experimental greenhouse vineyard, foliar diseases such as leaf spot, downy mildew, and sooty blotch occurred, along with cluster diseases including gray mold, ripe rot, sour rot, and *Aspergillus*. Among these, leaf spot, gray mold, and *Aspergillus* were the more severe diseases observed in the clean tillage treatment. These taxa often overwinter on fallen leaves in saprophytic or dormant states, serving as primary inoculum sources for infections in the following spring [8,34,35,36]. However, none of these pathogenic groups were detected in sheep fecal samples, indicating that ruminant ingestion and digestion act as an efficient biological barrier, effectively blocking the direct transmission pathway of phyllosphere fungal communities into the vineyard ecosystem.

Thus, sheep grazing can serve as a low input sanitation practice that not only removes fallen leaves but also reduces the reservoir of pathogens in vineyards, thereby lowering the inoculum pressure for foliar and cluster diseases. Importantly, compared to leaves, sheep feces showed a reduction in pathogenic bacteria such as *Erwinia* spp., while also introducing a substantial community of highly efficient cellulose degrading anaerobic gut bacteria into the vineyard system. These microbial communities may accelerate nutrient cycling and enhance soil fertility [16]. Furthermore, the input of a diverse and functionally distinct bacterial community could stimulate soil microbial activity and alter the competitive landscape in the grape rhizosphere. In general, an active and diverse soil microbiome can contribute to broad spectrum disease suppression through resource competition and niche exclusion [37]. In summary, using sheep to clear grapevine leaf litter in vineyards may not only eliminate potential pathogenic microorganisms but also generate multiple beneficial ecological effects.

Functional prediction analysis further supports the aforementioned shifts. The fecal bacterial community showed significant enrichment in fundamental metabolic pathways such as carbohydrate metabolism, membrane transport, and genetic information processing, which aligns with its adaptation to organic-rich anaerobic environments characterized by rapid heterotrophic growth and efficient nutrient acquisition [38]. In contrast, the leaf litter bacterial community was enriched in pathways associated with secondary metabolism (e.g., terpenoid and polyketide synthesis) and xenobiotic degradation, reflecting its adaptation to the specific phyllosphere environment, including challenges such as UV exposure, desiccation, and plant defense compounds [39]. Fungal functional prediction results indicated that, compared to leaf litter, sheep feces contained no fungi classified under the pathotroph trophic mode, further demonstrating that sheep grazing can reduce the input of pathogenic fungi into the vineyard system, thereby promoting vineyard health.

Sanitation practices are among the key vineyard management strategies. Traditional agricultural methods such as burial, removal, and burning aim to eliminate primary inoculum sources. However, introducing sheep into vineyards during winter not only effectively clears fallen leaves and reduces labor costs but also substantially mitigates the risk of fungal disease carryover via crop residues. Additionally, this practice may enhance nutrient cycling within the vineyard and promote healthier grapevine growth.

## 5. Conclusions

In conclusion, our results revealed that sheep grazing in greenhouse vineyards significantly reduced the incidence of leaf spot, sooty blotch, gray mold, sour rot, and *Aspergillus*. Notably, the suppressive effects of this practice on downy mildew and sooty blotch exhibit an ecological cumulative effect with prolonged treatment duration. Moreover, a fundamental difference in microbial composition was observed between fallen leaves and sheep feces, with no fungal taxa detected in fecal samples. Bacterial diversity was significantly higher in feces than in leaves. Grazing notably reduced grapevine pathogens while enriching bacterial groups potentially beneficial for vineyard nutrient cycling. Functional analysis revealed that fecal samples were enriched in pathways related to core metabolism, energy conversion, and genetic information processing, whereas leaf samples were associated with microbial interactions and complex secondary metabolism. Importantly, pathogenic fungal functional groups were absent in feces compared to fallen leaves. These results confirm that sheep grazing in vineyards significantly reduces the occurrence of grape diseases, winter sheep grazing effectively removes fallen leaves, reduces pathogen transmission risk, and may yield positive ecological benefits. This study provides novel information regarding the effects of sheep grazing in vineyards on pathogen suppression and grapevine health and supports the advancement of sustainable viticulture.

## Data Availability

The raw amplicon sequencing data for all samples involved in this study have been deposited at the National Center for Biotechnology Information (NCBI) under the accession number PRJNA1416020.

## Supplementary Materials

The following supporting information can be downloaded at: https://www.mdpi.com/article/10.3390/plants15060864/s1, Figure S1. Original electrophoresis gel image of fungal PCR products from sheep feces. The figure displays electrophoresis results of two replicates for seven sheep fecal samples, with a total of fourteen lanes corresponding to the upper twelve lanes and the two leftmost lanes in the lower row. Figure S2. Original electrophoresis gel image of fungal PCR products from grape fallen leaf. The upper section of the figure displays the electrophoresis results of fungal PCR products from seven fallen leaf samples.
