# Supplementary material for: Winter Grazing in Vineyards Suppresses Pathogens and Promotes Grapevine Health
Source: Plants (Basel). 2026 Mar 11;15(6):864. doi: 10.3390/plants15060864 (PMC13029377; doi:10.3390/plants15060864)
Supplement: Supplementary file 1 [file plants-15-00864-s001.zip › plants-4184773-supplementary.pdf]

Winter Grazing in Vineyards Suppresses Pathogens and Promotes Grapevine Health

Shaowei Cui [1,2], Lianzhu Zhou [2], Dong Li [1], Yanni Song [1], Hui Wu [2], Xiaoqing Huang [2], Decai Jin [3], Haijun Xiao [1,*], Yongqiang Liu [2,*]

1 School of Grassland Science, Beijing Forestry University, Beijing 100083, China

2 State Key Laboratory for Biology of Plant Diseases and Insect Pests, Institute of Plant Protection, Chinese Academy of Agricultural Sciences, Beijing 100193, China

3 Research Center for Eco-Environmental Sciences, Chinese Academy of Sciences, Beijing 100085, China

[*] Corresponding authors.
E-mail addresses: hjxiao@bjfu.edu.cn (H. Xiao), liuyongqiang@caas.cn (Y. Liu).

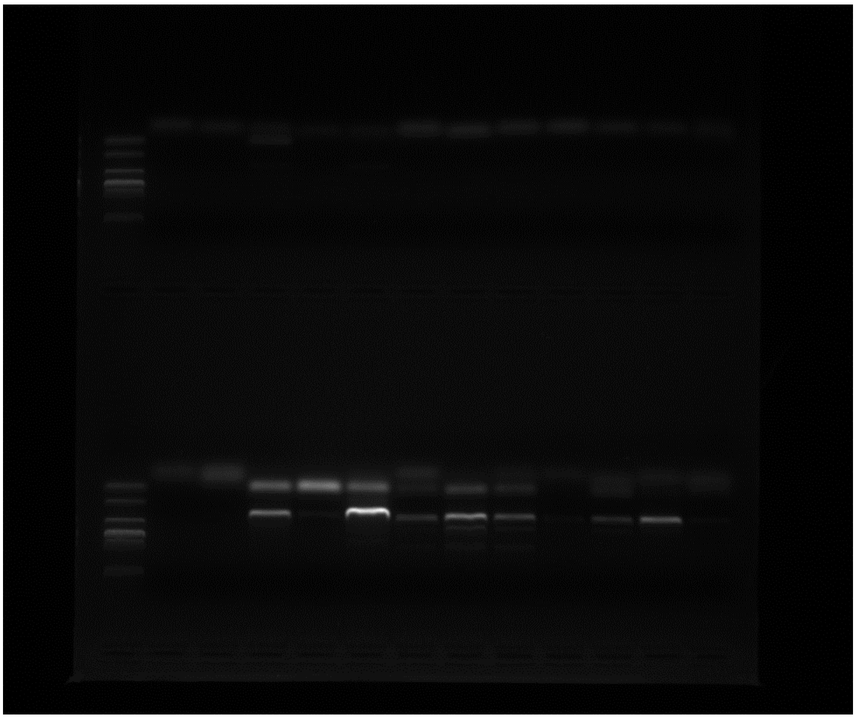

Figure S1. Original electrophoresis gel image of fungal PCR products from sheep feces. The figure displays electrophoresis results of two replicates for seven sheep fecal samples, with a total of fourteen lanes corresponding to the upper twelve lanes and the two leftmost lanes in the lower row.

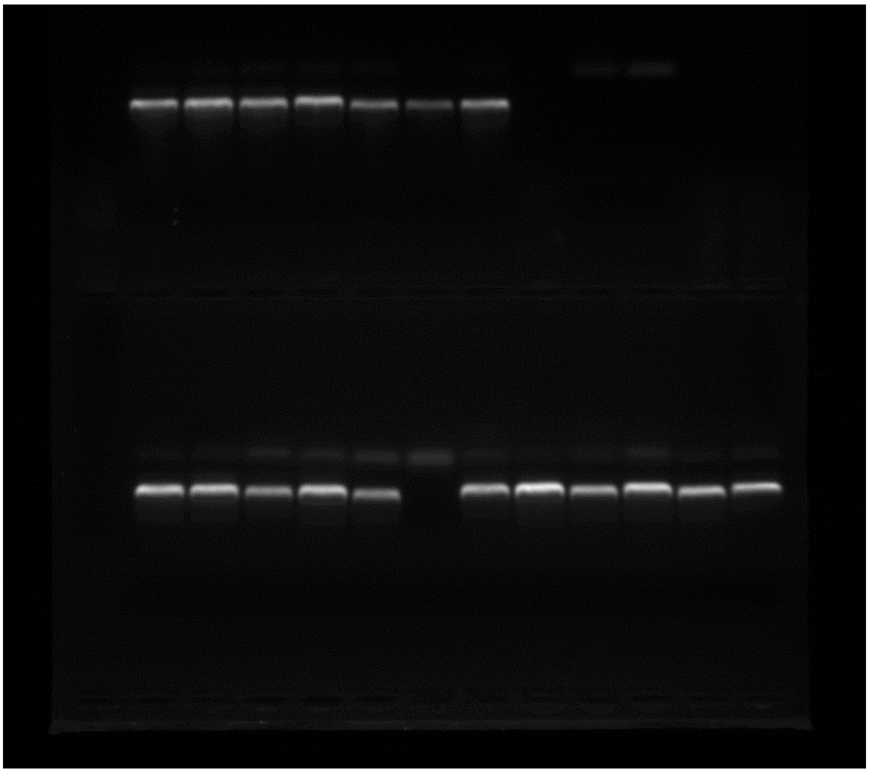

Figure S2. Original electrophoresis gel image of fungal PCR products from grape fallen leaf. The upper section of the figure displays the electrophoresis results of fungal PCR products from seven fallen leaf samples.
